# Pre- and during- labour predictors of dystocia in active phase of labour: a case-control study

**DOI:** 10.1186/s12884-020-03113-5

**Published:** 2020-07-28

**Authors:** Jila Nahaee, Fatemeh Abbas-Alizadeh, Mojgan Mirghafourvand, Sakineh Mohammad-Alizadeh-Charandabi

**Affiliations:** 1 Students’ Research Committee, Faculty of Nursing and Midwifery, Tabriz University of Medical Sciences, Tabriz, Iran; 2 Women’s Reproductive Health Research Center, Tabriz University of Medical Sciences, Tabriz, Iran; 3 Department of Midwifery, Faculty of Nursing and Midwifery, Tabriz University of Medical Sciences, Tabriz, Iran; 4 Social Determinants of Health Research Center, Department of Midwifery, Faculty of Nursing and Midwifery, Tabriz University of Medical Sciences, Tabriz, Iran

**Keywords:** Childbirth, Labour dystocia, Risk factor, Predictor, Iran

## Abstract

**Background:**

Labour dystocia (LD) is associated with maternal and foeto-neonatal complications and increased rate of caesarean section. There are scant studies on predictive factors of labour dystocia in Iran, as well as in other countries. Therefore, this study aimed to identify the predictive factors of LD using an integrated and collaborative pre- and during- labour factors to help formulate more effective intervention strategies for prevention and management of LD.

**Methods:**

In this case-control study, 350 women with and 350 women without LD, matched individually in terms of parity and hospital, were compared. The participants were in active labor, had singleton pregnancy, live foetus with a cephalic presentation, gestational age of 37^+ 0^–41^+ 6^ weeks, and were hospitalized for vaginal birth in two teaching hospitals in Tabriz, Iran. Data related to the socio-demographic characteristics, anxiety status (using the Spielberger State Anxiety Inventory), and woman dehydration were collected at cervical dilatation between 4 and 6 cm (before dystocia detection) and the other data at different phases of labour, and after birth (before discharge). The multivariate logistic regression was used to determine the predictors.

**Results:**

The predictors of LD were severe [OR 58.0 (95% CI 26.9 to 125.1)] and moderate [8.6 (4.2 to 17.4)] anxiety, woman dehydration > 3 h [18.67 (4.0 to 87.3)] and ≤ 3 h [2.8 (1.7 to 4.8], insufficient support by the medical staff in the delivery room [5.8 (1.9 to 17.9)], remifentanil administration [3.1 (1.5 to 6.2)], labour induction [4.2 (2.5 to 7.2], low income [2.0 (1.2 to 3.3)], woman’s height < 160 cm [2.0 (1.1 to 3.3)], and woman age of 16–20 y [0.3 (0.2 to 0.6)]. The proportion of the variance explained by all these factors was 74%.

**Conclusion:**

The controllable predictors, such as woman anxiety and dehydration, and insufficient support from medical staff during labour were strongly associated with the risk of LD. Therefore, it seems that responding to woman physical, psychological, and supportive needs during labour can play a significant role in LD prevention and control.

**Ethical code:**

IR.TBZMED.REC.1397.624.

## Background

Labour dystocia (LD) is defined as slow progress of labour or the lack of progressive cervical dilatation, and/or no descent of the foetal head [1]. It can result in maternal and foeto-neonatal complications, such as infection, postpartum bleeding, foetal distress, increased rate of caesarean section (CS), and mortality, as well as unpleasant childbirth experience [2–4]. Therefore, timely diagnosis of the problem and its management are very important [3].

The reported incidence of LD is 7.5% in Qom-Iran [5], 12.1% in Mashhad-Iran [6], 37% in Denmark [2], and 30% in Sweden [7]. This significant difference in the incidence is probably due to difference in diagnostic methods.

Various degrees of increase in CS rate have been observed in different countries across the world in the past 30 years. LD has been reported as the leading cause of increase in the CS rate in the US and Canada [8]. It is estimated that LD accounts for approximately 60% of all CS operations [9]. According to a review, the incidence of CS is 48% in Iran [10]. A study in Sanandaj-Iran reported that 33.5 and 24.5% of CS cases in 2005 and 2009 were due to LD, respectively [11].

There are relatively scant studies on the risk, especially on predictive factors of labour dystocia in Iran [5, 6] and other countries [12–16]. These studies reported primiparity, labour induction, premature rupture of membrane (PROM), hypertensive disorders, poly-hydramnios, older maternal age [12], occiput posterior foetal position [15], and foetal macrosomia [16] as the risk factors of LD. However, in most of the previous studies, only a few number of possible risk factors have been investigated and we found no study investigating simultaneously almost all possible pre- and during- labour risk factors of LD and their predictive power. Also, previous studies reported epidural analgesia as a risk factor of LD [13, 14]; but, we found no study assessing the effect of remifentanil, an opioid analgesic drug which are commonly used in our settings, on LD. Therefore, considering the lack of sufficient evidence, we aimed to determine the risk and predictive factors of LD using an integrated and collaborative pre- and during- labour factors to help formulate more effective intervention strategies for prevention and management of LD.

## Methods

### Study design and setting

This is a case-control study, in which the participants were individually matched in terms of parity (primiparity and multiparity) and hospital. Data were collected from Oct 2018 to Jul 2019 at two teaching hospitals (Taleghani and Al-Zahra), affiliated to the Tabriz University of Medical Sciences.

Tabriz, the capital city of East Azerbaijan province, is a mountainous region in north-west of Iran with Azari speaking people and a population of about 1,770,000. Taleghani is a tertiary hospital for referral from other centres in Tabriz and provincial cities and Al-Zahra is a tertiary hospital for referral from other centres in Tabriz and provincial cities, and also from Ardabil, West Azerbaijan, Kordestan, and Zanjan provinces with a total population of 5,200,000. The childbirth rate in each of these hospitals is about 500 births per month.

Birth attendants at the two hospitals are almost the same. Almost all of the deliveries were attended by students (gynaecology residents, interns or midwifery students) who work periodically in the both hospitals; only less than 5% of the deliveries were attended by the midwives who were staff of the hospitals. The gynaecology residents had direct responsibility for examinations and prescriptions during labour and delivery.

In these hospitals, an intravenous cannula is inserted for all women at admission to the delivery room for keeping vein open; however, Ringer’s solution infusion is commenced only after the physician orders for labour induction or augmentation. At any labour stages, oxytocin, amniotomy, and analgesics (hyoscine, promethazine, pethidine, or combination of two or three of them) could be administered by the gynaecology resident to deal with maternal agitation and facilitate the progression of dilation under slow labour progression conditions. Remifentanil was administered in case of normal foetus cardiogram and a minimum cervical dilatation of 5 cm, regardless of LD, after obtaining the informed written consent from parturients. The foetal heart and uterine contractions were regularly monitored with tococardiogram devices and parturients could mobilise out of the bed for a short period of time. The participants had access to food and/or drink (based on the stage of labour); however, since they were lying on the bed and could not have a companion, and there was not enough staff, they were not given adequate liquids. In every shift, one midwife was responsible for caring two to three parturients, executing resident’s orders, and controlling foetal heart and uterine contraction.

The inclusion criteria were primiparous or multiparous women in active labor with cervical dilation of 4–6 cm, regular uterine contractions, and normal tococardiography at admission, those with a history of one or two vaginal deliveries, gestational age of 37^+ 0^–41^+ 6^ weeks, singleton pregnancy, live foetus with a cephalic presentation. Those with failed inductions were excluded. The other exclusion criteria were women with psychological, speech, hearing, visual and mental disorders, genital infection not allowing vaginal delivery, such as genital herpes, untreated fever with unknown pathology, abnormalities in pelvic size based on vaginal examination (ischial spine, obstetrical conjugate, concavity of sacrum, sacral promontory and pubic angle), abnormal bleeding, any abnormality in soft or bone tissues of genital area, and elective CS.

### Data collection

Although the hospitals have guidelines for definition of active labor, for labor dystocia and how and when to intervene in case of dystocia, the guidelines were not followed exactly most of the times and medical records were not complete. Therefore, selection of participants and collection of all data for this study was done by the PhD midwifery student (first author) who is highly experienced person in vaginal childbirth.

Potentially eligible women in the labour unit were selected after completing the eligibility checklist through interview and vaginal examinations. The initial vaginal examination was done to determine dilation, effacement, descent of the foetal head, amniotic membrane condition (tact/intact), and pelvic condition for any abnormality. The socio-demographic questionnaire, state anxiety inventory and dehydration checklist were completed before detection of LD, in cervical dilatation of 4–6 cm.

The socio-demographic questionnaire (Additional file 1) was completed using the medical records, clinical examination, and interview with the women. The data extracted from medical records of the participants were age, parity, and pre-pregnancy weight. In case of no prenatal care records, the aforementioned data were collected by asking the participants. Data related to height and weight were measured via clinical examinations using a stadiometer and a digital scale, respectively. Data related to family income, educational attainment, and job of the participant and her spouse, intended or unintended pregnancy, foetal sex preference, participation in childbirth educational classes, active or passive tobacco smoking, alcohol consumption, emotional, physical, and sexual violence during pregnancy, and preference of the participant and her spouse for birth method was collected through interview. The body mass index (BMI) was calculated by dividing weight (kg) by square of the height (m).

Pre-pregnancy BMI <  18.5 was regarded as low weight, 18.5 to 24.9 as normal weight, 25 to 29.9 as overweight, and >  30 as obese. According to the US institute of medicine (IOM), normal weight gains during pregnancy based on the BMI were regarded as 12.5–18, 11.5–16, 7–11.5, and 5–9 kg, respectively [17] .

Exposure to more than two cigarettes per day, on average, during the pregnancy was regarded as passive smoking. Experience of emotional, physical and sexual violence during pregnancy was assessed with three questions (one for each of them) with three “never,” “sometimes,” and “most often,” options. During the analysis of responses, the sometimes and most often responses were unified.

The Spielberger’s State Anxiety Inventory was used to measure anxiety. It is a 20-item self-report questionnaire with minimum and maximum total scores of 20–80. Each item is scored by 1 “very low,” 2 “low,” 3 “high,” and 4 “very high.” Some items are scored inversely (1, 2, 5, 8, 10, 11, 15, 16, 19, and 20). Finally, the total score is regarded as the score of anxiety. Scores 20–40 are regarded as mild anxiety, 41–54 as moderate anxiety, and ≥ 55 as severe anxiety [18].

Dehydration was diagnosed by examining the following signs and symptoms: dry mouth and lips, thirst, dizziness or weakness (despite normal blood pressure), difficult speaking due to a sticky dry feeling in the mouth, and difficult swallowing [19]. Each of these symptoms or signs was enough for diagnosis of dehydration. Duration of dehydration, if any, was asked from the participants. Those dehydrated cases that lasted less than 30 min were regarded as “No.”

The labour progression checklist (Additional file 2) was completed during the labour stages until the completion of delivery through observation or clinical examinations. Vaginal examinations were repeated every four hours after the initial examination and even sooner, if needed (e.g. frequent regular contractions as a sign of second stage of labour). Based on the Zhang’s guideline, participants with abnormal and normal progression of labour were placed in the “case” and “control” groups. For every participant in the case group, her following matched parturient with no LD was placed in the control group.

LD was diagnosed based on the Zhang’s guideline, approved by the American Consortium on Safe Labour [20]: Slow progression in cervical dilatation from 4 cm to 5 cm in ≥6 h, progression of cervical dilation from 5 cm to 6 cm in ≥3 h (in both primiparous and multiparous women), and progression of cervical dilation < 0.5 cm per hour for primiparous women and < 0.7 cm per hour in multiparous women at 6 cm cervical dilation in two hours. The second stage > 2.8 h in primiparous women and > 1 h in multiparous women without epidural analgesia was regarded as the signs of LD (there was no case receiving epidural analgesia among the samples).

Data about other during-labour interventions including augmentation, and administration of analgesic and remifentanil were collected via observation during labour by the investigator.

Two subscales of the Mackey Childbirth Satisfaction Rating Scale [21] (satisfaction with the doctor and nurse-midwife, items 17–33) were used to measure the support from nurse-midwife and physician. Twelve to twenty-four hours after the birth, before discharge, the researcher attended the postpartum unit to complete the Mackey questionnaire through interview.

The foetal-neonatal checklist (Additional file 3) was completed at enrolment and during labour stages (using cardiography) and after childbirth (neonatal weight, height, and head circumference).

The content validity was used to validate the socio-demographic questionnaire, labour progression checklist, and foetal-neonatal checklist during labour, and postpartum foetal-neonatal checklist. The internal consistency of the state anxiety and two subscales of the Mackey Childbirth Satisfaction Rating Scale, namely satisfaction with the doctor and nurse, was confirmed in this study and their Cronbach’s alpha were 0.94, 0.97, and 0.91, respectively.

### Statistical analysis

Using the method proposed by Riley et al. [22], based on up to 10 candidate predictor parameters with an anticipated adjusted Nagelkerke’s R^2^ of at least 0.25, to target an expected shrinkage of 0.95, we needed a minimum sample size of 655. We considered 700 samples (350 for each case and control groups) in this study.

The statistical analysis was done using SPSS 21 (SPSS, Chicago, IL, USA). To ensure that no selection bias was introduced, we compared the women with no dystocia who selected as control group with those not selected as control group in terms of some background characteristics using ANCOVA for continuous factors or logistic regression for categorical factors, adjusted for the matching factors (parity and hospital).

The association of each pre- and during-labour variable with LD was identified using the binary logistic regression, adjusted for the matching factors. Variables with *p* < 0.2 in the primary analysis were entered into the multivariable binary logistic regressions using the backward LA strategy and three dystocia prediction models were developed for following factors by controlling different factors: per-labour factors, during-labour factors, and overall factors. The goodness of fit of the models was investigated using the Hosmer and Lemeshow test. The Nagelkerkes R square was used to measure the proportion of total variance predicted by the models. In this study, *p* < 0.05 was considered to be significant.

## Results

**Fig. 1:**
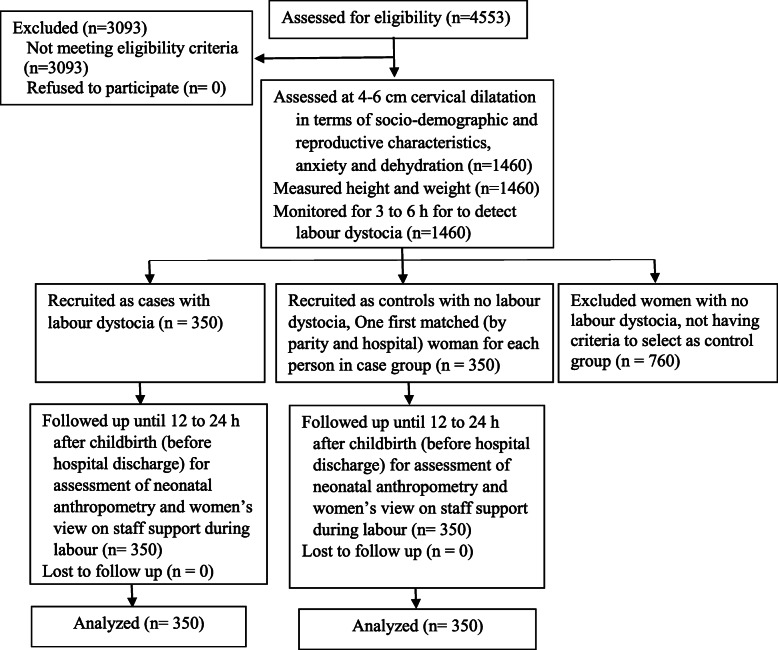
Study flow diagram

There was no eligible person refusing to participate in the study. Data at recruitment were collected from 1460 women, of which 350 women were diagnosed with dystocia and considered as case group. As we planned to recruit only one first matched control for each person in case group, we did not follow up the other 760 women with no dystocia, after delivery. All 350 participants in each group were followed up until hospital discharge and there was no missing value for essential data (Fig. 1).

Comparison of the 350 women in the control group with the 760 women not selected as controls, adjusted for parity and the participating hospital, showed no significant differences between the groups in terms of the background factors (*P* > 0.05), except mean of gestational age. Although mean gestational age of the study control group was significantly higher than the non-selected group (39.2 vs 39.0 weeks, *P* = 0.003) but when we compared the groups in terms of the gestational age category, as we used in this study (37^+ 0^ to 39^+ 6^, 40^+ 0^ or more), the difference was not significant (*P* = 0.555).

Out of the 700 participants, 354 were from Al-Zahra Hospital and 57% were primiparous women. None of the participants reported the use of hookah or alcohol during their pregnancy. Only one participant in the case group reported tobacco smoking during her pregnancy. Almost all (98%) of the participants were housewives. Experience of sexual violence during pregnancy was reported in 17% of participants in the dystocia group and 5% of participants in the control. CS was observed in 12% of the participants in the case group and none of the participants in the controls. Two case and one control participants had poly-hydramnios; 14 case and 7 control participants had pregnancy-induced hypertension; 34% of case participants and 7% of controls had PROM; 4 case and 3 control participants had persistent occiput posterior position; 6% of case neonates and 3.4% of control neonates had a birth weight of > 4000 g; 98% of case participants and 70% of the controls received analgesics. In the control group, there were four foetus with cardiac late deceleration that with some measures for the women (like discontinuing oxytocin infusion, oxygen infusion, hydration and changing position to left side), their foetal cardiogram returned to normal. One neonate in the case and none in the control group had Apgar score of < 7 at the fifth minute.

### Association of pre- and during-labour factors with LD

The following factors were significantly associated with increased odds of LD: gestational age ≥ 40 w; woman’s height < 160 cm; history of emotional, physical, or sexual violence during pregnancy; low household income; woman preference for CS (during pregnancy); obesity; excessive gestational weight gain; exposure to tobacco smoke during pregnancy; not participating in labour education classes; woman dehydration during labour; labour induction; anxiety during labour; receiving remifentanil; insufficient support from medical staff in the delivery room. The age group of 16–20 y and weight gain lower than normal level were significantly associated with reduced odds of LD (Table 1).

**Table 1:** Association of some demographic, obstetrics and during labour variables with labour dystocia

	Case (*n* = 350)	Control (n = 350)	Odds Ratio (95% CI)	P
Age (year)				
13–15	3 (0.9%)	3 (0.9%)	0.92 (0.18 to 4.62)	0.923
16–20	68 (19.4%)	92 (26.3%)	0.68 (0.47 to 0.98)	0.040
21–34	235 (67.1%)	217 (62.0%)	reference	
+ 35	44 (12.6%)	38 (10.9%)	1.07 (0.67 to 1.71)	0.781
Gestational age (week)				
37^+ 0^ to 39^+ 6^	200 (57.1%)	244 (69.7%)	reference	
40^+ 0^ or more	150 (42.9%)	106 (30.3%)	1.73 (1.26 to 2.36)	0.001
Woman^’^s height < 160 cm	189 (54%)	105 (30.0%)	2.74 (2.01 to 3.74)	< 0.001
Violence during pregnancy				
Physical violence	41 (11.7%)	20 (5.3%)	2.19 (1.26 to 3.83)	0.006
Emotional violence	126 (35.8%)	67 (19.1%)	2.36 (1.67 to 3.33)	< 0.001
Sexual violence	59 (16.9%)	16 (4.6%)	4.25 (2.39 to 7.54)	< 0.001
Low household income	216 (61.7%)	164 (46.9%)	1.82 (1.35 to 2.46)	< 0.001
Woman preference for CS (during pregnancy)	66 (18.9%)	34 (9.7%)	2.16 (1.39 to 3.37)	0.001
Pre-pregnancy BMI (kg/m^2^)				
Low weight (< 18.5)	13 (3.7%)	19 (5.4%)	0.86 (0.41 to 0.79)	0.686
Normal weight (18.5–24.9)	144 (41.3%)	182 (53%)	reference	
Over weight (25–29.9)	133 (38.1%)	124 (35.4%)	1.35 (0.97 to 1.87)	0.075
Obesity (> 30)	59 (16.9%)	25 (7.1%)	2.96 (1.77 to 4.96)	< 0.001
Gestational weight gain^a^				
Normal	122 (34.9%)	127 (36.3%)	reference	
Over normal	169 (48.3%)	99 (28.3%)	1.79 (1.26 to 2.55)	0.001
Less than normal	59 (16.9%)	124 (35.4%)	0.49 (0.34 to 0.74)	0.001
No participating in labour education classes	323 (92.6%)	303 (86.6%)	1.76 (1.07 to 2.92)	0.027
Exposure to tobacco smoke during pregnancy^b^ (Passive smoker vs. no smoker)	122 (34.7%)	53 (15.1%)	2.97 (2.06 to 4.29)	< 0.001
Insufficient support by staff ^c^	107 (30.7%)	4 (1.1%)	38.60 (14.04 to 106.14)	< 0.001
Anxiety levels^d^				
Mild	14 (4.0%)	223 (63.7%)	reference	
Moderate	89 (25.4%)	97 (27.7%)	16.68 (8.91 to 31.23)	< 0.001
Severe	247 (70.8%)	30 (8.6%)	167.20 (83.53 to 334.68)	< 0.001
Labour induction	244 (69.7%)	56 (16.0%)	12.08 (8.38 to 17.42)	< 0.001
Analgesics administration	344 (98.3%)	244 (69.7%)	0.04 (0.02 to 0.09)	< 0.001
Remifentanil administration (pain killer)	79 (22.6%)	39 (11.1%)	2.32 (1.53 to 3.53)	< 0.001
Woman dehydration^e^				
No	113 (32.4%)	252 (72.0%)	reference	
Yes, ≤ 3 h	181 (51.6%)	95 (27.1%)	4.25 (3.04 to 5.93)	< 0.001
Yes, > 3 h	56 (16.0%)	3 (0.9%)	41.63 (12.76 to 135.81)	< 0.001
Weight of neonate (g)				
2500 to 3499	223 (63.7%)	253 (72.3%)	reference	
Less than 2499	5 (1.4%)	9 (2.6%)	−0.46 (0.21 to 1.91)	0.414
> 3500	122 (34.9%)	88 (25.1%)	1.57 (1.13 to 2.18)	0.007
Head circumference of neonate > 35 cm	126 (36.0%)	69 (19.7%)	2.29 (1.63 to 3.2)	< 0.001
Height of neonate > 52 cm	76 (21.7%)	49 (14.0%)	1.70 (1.15 to 2.53)	0.008

All analysis were done using binary logistic regression adjusted for parity (primi- or mulity-parity) and hospital (matching factors)

^a^ Normal weight gain (kg) during pregnancy based on pre-pregnancy BMI: less than 18.5 kg/m^2^: 12.5–18, between 18.5 and 24.9 kg/m^2^: 11.5–16, between 25 and 29.9 kg/m^2^: 7–11.5, greater than 30 kg/m^2^: 5–9 [17]; ^b^There was only one active smoker and daily expose to more than 2 cigarettes was considered as positive exposure; ^c^Assessed by nurse and physician subscale of Mackey satisfaction tool (range score: 17–85): score 17–51 (low satisfaction), score 52–85 (good satisfaction) [21]; ^d^Spielberger anxiety score (range score: 20–85): 20–40 (mild), 41–54 (moderate), > 55 (severe) [18]; ^e^existence of at least one of dehydration signs or symptoms (dry mouth and lips, thirst, dizziness, weakness, trouble swallowing dry food, dry, sticky mouth that makes it hard to talk, a swollen, cracked or dry tongue) [19]

70% of cases had inductions of labor compared to 16% of controls. The reasons for the induction in the groups were: PROM (49.6% vs 42.9%), early hospitalization before the onset of spontaneous labour contractions (46.3% vs 44.6%), complications such as high blood pressure, oligohydramnios, thrombocytopenia, nephropathy (4.1% vs 12.5%).

Foetal height > 52 cm, head circumference >  35 cm, and birth weight >  3500 g were significantly associated with increased odds of LD. However, since their pre-labour measurement was challenging, these variables were not inputted into the regression model to determine the predictors (Table 1).

The Hosmer and Lemeshow test showed that all three prediction methods (pre-labour characteristics, during-labour factors, and overall factors) fitted data well (*p* > 0.1).

### Pre-labour predictors of LD

The most important predictors included woman’s height < 160 cm, sexual abuse, exposure to tobacco smoke during pregnancy, and woman pre-labour preference for CS. Proportion of the variance explained by all the pre-labour factors was 25% (Table 2).

**Table 2:** Pre- and during- labour predictors of labour dystocia

Predictors	AOR (95% CI)	P
1. Pre-labour predictors^*^		
Low household income	1.46 (1.04 to 2.05)	0.027
Woman height < 160 cm	2.75 (1.96 to 3.85)	< 0.001
Exposure to tobacco smoke during pregnancy (Passive smoker vs. no smoker)	2.23 (1.50 to 3.32)	< 0.001
Sexual violence during pregnancy	2.36 (1.26 to 4.39)	0.007
Weight gain during pregnancy (Ref: normal)		
Over normal	1.65 (1.13 to 2.42)	0.010
Less than normal	0.51 (0.33 to 0.79)	0.002
Woman preference for CS (during pregnancy)	2.12 (1.29 to 3.47)	0.003
Gestational age of 40^+ 0^–41^+ 6^ w (Ref: 37^+ 0^–39^+ 6^)	1.75 (1.24 to 2.47)	0.002
**2. During labour predictors**†		
Labour induction	4.31)2.61 to 7.11)	< 0.001
Anxiety (Ref: mild)		
Moderate	8.45 (4.33 to 16.49)	< 0.001
Sever	52.54 (25.61 to 107.79)	< 0.001
Woman dehydration (Ref: no)		
3 h or less	2.43 (1.47 to 4.00)	0.001
More than 3 h	15.41 (3.77 to 63.04)	< 0.001
Remifentanil administration (pain killer)	2.59 (1.32 to 5.09)	0.006
Insufficient support by staff	7.24 (2.40 to 21.78)	< 0.001
**3. All variables**^**‡**^		
Woman age of 16–20 (Ref: 21–34 year)	0.32 (0.17 to 0.61)	< 0.001
Woman height < 160 cm	1.93 (1.14 to 3.27)	0.014
Low household income	1.94 (1.16 to 3.25)	0.012
Labour induction	4.23 (2.48 to 7.22)	< 0.001
Remifentanil administration (pain killer)	3.09 (1.53 to 6.22)	0.002
Woman dehydration (Ref: no)		
Yes, ≤ 3 h	2.85 (1.68 to 4.83)	< 0.001
Yes, > 3 h	18.67 (3.99 to 87.27)	< 0.001
Anxiety (Ref: mild)		
Moderate	8.56 (4.21 to 17.41)	< 0.001
Sever	58.03 (26.91 to 125.14)	< 0.001
Insufficient support by staff	5.75 (1.85 to 17.93)	0.003

*AOR* adjusted odds ratio; All analysis were done using binary logistic regression with backward (LA) variable selection

* Adjusted for all other pre-labour variables with a relation of p < 0.2 in the primary analysis, and parity (primi- or mulity-parity) and hospital (matching factors), variables of attendance at pregnancy classes, physical and emotional abuse, pre-pregnancy BMI, woman age were removed from the model. *P* = 0.701 for Hosmer & Lemeshow test of the goodness of fit, Nagelkerkes R^2^ = 0.25

† Adjusted for all during labour variables with a relation of *p* < 0.2 in the primary analysis, and parity (primi- or mulity-parity) and hospital (matching factors). No variables were removed from the model. *P* = 0.205 for Hosmer & Lemeshow test of the goodness of fit, Nagelkerkes R^2^ = 0.71

‡ Adjusted for all variables entered in the above models. *P* = 0.640 for Hosmer & Lemeshow test of the goodness of fit, Nagelkerkes R^2^ = 0.74

### During-labour predictors of LD

These predictors included severe [52.5 (25.6 to 107.8)] and moderate anxiety [8.5 (4.3 to 16.5)], woman dehydration > 3 h [15.4 (3.8 to 63.0)] and ≤ 3 h [2.4 (1.5 to 4.0)], insufficient support by staff [7.2 (2.4 to 21.8)], induction of labour [4.3 (2.6 to 7.1)], and receiving remifentanil [2.6 (1.3 to 5.1)]. The proportion of the variance explained by these factors was 71% (Table 2).

### Overall predictors of LD

These predictors were severe [58.0 (26.9 to 125.1)] and moderate [8.6 (4.2 to 17.4)] anxiety, woman dehydration > 3 h [18.67 (4.0 to 87.3)] and ≤ 3 h [2.8 (1.7 to 4.8)], insufficient support from medical staff in the delivery room [5.8 (1.9 to 17.9)], remifentanil administration [3.1 (1.5 to 6.2)], labour induction [4.2 (2.5 to 7.2)], low household income [2.0 (1.2 to 3.3)], woman’s height < 160 cm [2.0 (1.1 to 3.3)] and woman age of 16–20 y [0.3 (0.2 to 0.6)]. The proportion of the variance explained by all these factors was 74% (Table 2).

## Discussion

During-labour predictors of LD, comprising woman dehydration, anxiety, insufficient support from medical staff, labour induction, and remifentanil administration, explained higher proportion of the variance compared to the pre-labour predictors (71% vs 25%). The overall predictors of LD were woman dehydration; moderate and severe anxiety; labour induction; low household income; remifentanil administration; insufficient support from the medical staff; and woman’s height < 160 cm. The age group of 16–20 years was a protective factor.

In this study, gestational age ≥ 40 weeks increased the risk of LD by 1.75 times. In studies in Israel [12] and Sweden [13], the frequency of LD was also significantly higher in gestational age ≥ 42 weeks. Also, our results on excessive gestational weight gain as a predictor of LD is consistent with a study results in the US [23]. An increase in foetal weight, height, and head circumference with increasing the gestational age, also with excessive gestational weight gain may be important contributing factors resulting in slow progress of labour. In the present study, the feotal weight >  3500 kg, height > 52 cm, and head circumference >  35 cm were associated with a significant increase in the odds of dystocia.

Woman preference for CS before the onset of labour as a predictor of LD is probably due to high level of maternal fear of labour in these women. In a study in Denmark, fear increased the odds of LD by 1.33 times [24]. It is expected that fear negatively affects uterine contractions by increasing catecholamines [25].

Low household income as a risk factor of LD in our study is consistent with results of a study in Uganda [26]. This association may be related to the higher rate of obesity among Iranian women with lower economic status [27]., and/or high level of stress and anxiety during pregnancy and labour due to such factors as worry over the cost of raising a child in such families.

In the present study, sexual violence was among the pre-labour predictors of LD, although it was removed in the overall model. These results are consistent with other studies in Iran (Khorram-abad) [28] and in Iceland [29]. But a study in Denmark showed no relationship between violence during pregnancy and prolonged labour [30]. This inconsistency may be due to the low frequency of sexual violence during pregnancy (2.5%) and probable low chronicity and severity in the Danish study.

In this study, exposure to tobacco smoke during pregnancy was a pre-labour predictor of LD; however, it was removed in the overall model of predictors. A study in England also showed a significant relationship between maternal smoking and LD [31]. It was shown that exposure to tobacco smoke could result in reduced blood supply and increased lactate in uterine capillaries. Accumulation of lactic acid in muscles could lead to an irregular contraction pattern which, in turn, result in insufficient uterine contractions during labour [32].

In addition, we found that the woman’s height < 160 cm is a risk factor of LD. A study in Denmark showed that the frequency of LD in people < 160 cm was significantly higher than in people > 170 cm; and there was no significant difference between groups with woman’s height 160–169 cm and those with height ≥ 170 cm [33]. In the present study, due to limited number of mothers with the height of > 170 cm (4.5%), we only compared those < 160 cm with those ≥160 cm.

We assessed existence of dehydration by examining the signs and symptoms and assessed dehydration duration only by asking the women and did not assess objectively the amount of liquids received by the women. However, the results on the woman dehydration as a risk factor of LD could be considered consistent with results of meta-analysis of the Cochrane systematic review on four trials (808 women) which indicates that in women with restricted oral intake, higher infusion rate of intravenous fluids significantly reduce the duration of labour [34]. It is probable that dehydration during labour reduces metabolic reservoirs, i.e. glycogen, in uterine muscles, maintains natural level of adenosine three phosphate (ATP) in disrupted myometrial muscles, and increases acidity which, in turn, disrupt the performance of uterine muscles [35].

In this study, anxiety, specifically severe woman anxiety during labour (at cervical dilation of 4–6 cm), was the most strong predictor of LD. A study in Oman showed similar results [36]. But, a study in Tehran-Iran did not show any significant relationship between moderate anxiety and duration of labour stages in primiparous women [37]. This difference can be due to the difference in time of anxiety measurement. In the study in Tehran, level of anxiety was measured at cervical dilation < 4 cm (in about half of cases at cervical dilation 2 cm) at admission to the labour room, before experience of pain at active phase of labour. Maternal anxiety during labour may reduce uterine contractions through increasing the level of epinephrine [25].

Supportive care is defined as providing emotional support, information, counselling services, and comfort during labour [38]. In our study, the odds of LD was 6.4 times in women receiving insufficient support from nurses/midwives, and physicians. A recent Cochrane review study including 13 trials showed that continuous care during labour significantly reduced labour duration, and the need for oxytocin and analgesics [39]. A study in China showed that the labour duration in women receiving routine care was two times compared to women receiving supportive care [40].

Previous studies showed that labour induction significantly increase the odds of LD even with controlling the woman’s weight, foetal weight, and gestational age [41, 42] which is consistent with the results of present study. This can be due to the low Bishop score at the onset of induction, resulting in a weak response to induction and prolonged labour (in the present study, approximately 70% of women in the LD group received induction).

In our study, the odds of LD increased by three times following remifentanil administration. It is an artificial opioid that directly affects the μ- opioid receptor and induces analgesia effect [43]. We did not find any other study on its effect on LD and also on the mechanism of its adverse effect on the labour duration.

The age group of 16–20 years as a protective factor of LD in our study can be considered consistent with the results of the studies in the US [44, 45]. A study reported significantly lower prevalence of LD-induced CS in women younger than 25 years old than women aged ≥40 years [44]; and another study showed that aging (> 25 years) increases the duration of the first and second labour phases and risk of LD-induced CS [45].

### Strengths and limitations

Among the strengths of this study was investigating the majority of dystocia risk factors, including woman anxiety and dehydration, before detection of dystocia. Dystocia was detected prospectively using an objective criterion. These can reduce the assessment bias. Relatively high sample size, specifically the LD cases, can be regarded as another strength of the present study, which allows for investigating more risk factors with higher accuracy. Nevertheless, studying the effects of some possible risk factors, such as primiparity (due to individual matching of the groups based on this variable), poly-hydramnios, hypertension, and persistent occiput posterior position (due to low number of these cases) on LD was not possible in this study. Moreover, we had no information about history of dystocia in previous deliveries, due to the lack of access to previous medical records of the multiparous women.

Some variables related to lifestyle, including nutrition and physical activity, may be associated with dystocia and/or interact with the factors under consideration in the present study. In this study, these variables were not assessed to prevent too much items to respond. However, this consideration can be regarded as a limitation of the present study.

In the study setting, early hospitalization before the onset of spontaneous labour pain at the 40th week (as demanded by the woman due to the issue of distance), early initiation of interventions, and hospitalization in general rooms that exposed the participants to the conditions of other parturients could increase the level of anxiety. Moreover, this study was conducted in a medicalized setting, which lacks most of requirements for physiological childbirth, such as no opportunity to walk or movements in bed freely; no access to childbirth equipment like birthing balls and warm douching; no program for respiration and relaxation training by the medical staff; no companion at bed in majority of cases. Therefore, results of this study may not be generalized into other settings which are woman-centred and are equipped well for physiological childbirth.

Regarding these limitations, it is recommended to conduct more observational studies with controlling the lifestyle of the participants in settings equipped well for physiological childbirth. Regarding the nature of observational studies, the obtained relationships cannot be considered as cause-and-effect. Therefore, it is recommended to perform interventional studies to determine the effect of the controllable factors, such as effect of method to relief anxiety and prevent maternal dehydration, as well as providing sufficient support from medical personnel during labour on labour dystocia.

## Conclusions

The controllable predictors, such as anxiety, maternal dehydration, and the lack of adequate support from medical staff during labour are strongly associated with the risk of LD. The pre-labour predictors determined in the current study, such as excessive weight gain during pregnancy, maternal preference for CS, sexual violence during pregnancy, and daily exposure to tobacco smoke, which may need more comprehensive and multifaceted interventions for their control, explained relatively low proportion of the variance. Therefore, it seems that responding to women’s physical, psychological, and supportive needs during labour could play a significant role in prevention and appropriate control of LD.

## Supplementary information

**Additional file 1.** Woman demographic characteristics sheet.

**Additional file 2.** Labour progress sheet.

**Additional file 3.** Neonate form.

## Data Availability

The datasets used and analysed during the current study are available from the corresponding author on reasonable request.

## Abbreviations

LD: labour dystocia
CS: caesarean section
BMI: body mass index
OR: odds ratio
CI: confidence interval
