# Supplementary material for: Pre- and during- labour predictors of dystocia in active phase of labour: a case-control study
Source: BMC Pregnancy Childbirth. 2020 Jul 28;20:425. doi: 10.1186/s12884-020-03113-5 (PMC7388514; doi:10.1186/s12884-020-03113-5)
Supplement: Supplementary file 1 — Additional file 1. Woman demographic characteristics sheet. [file 12884_2020_3113_MOESM1_ESM.docx]

Woman demographic characteristics sheet

Code: ……….

1. Date of birth: ……….
2. Age: ……….
3. Number of pregnancy: ……….
4. Number of childbirth: ……….
5. Number of alive child: ……….
6. History of abortion: no□ yes□,(Induced: ………. Spontaneous: ………. )
7. How much did pass from your last childbirth: ……….
8. Gestational age: by sonography: ………. by LMP: ……….
9. Woman education: primary□ secondary□ high school□ diploma□ university□
10. Husband education: primary□ secondary□ high school□ diploma□ university□
11. Woman occupation: household□ employed□ student□ other□
12. Husband occupation: no occupation□ employed□ freelance job□ other□
13. Did you want this pregnancy: no□ yes□
14. Did your husband want this pregnancy: no□ yes□
15. Did you attend at birth educational classes during this pregnancy: no□ yes□,

if yes, how many sessions; theoretical: ………. practical: ……….

1. If you attend at birth classes, how much was useful?

Very much□ much□ not much□ not useful□

1. Have you ever smoked during this pregnancy: no□ yes□

If yes, how many cigarette daily: ………. less than 1 cigarette□

1. Were you in a place where there was smoke: no□ yes□

If yes, how many cigarette daily: ………. sometimes□

1. Is your husband smoker: no□ yes□

If yes, how many cigarette daily: ………. less than 1 cigarette□

1. Is someone else regularly smoke where you live or work? no□ yes□

If yes, how many cigarette daily: ………. less than 1 cigarette□

1. How is your family income:

very less than enough□ less than enough□ enough□ more than enough□

1. Did you drink alcohol at pregnancy: no□ yes□
2. Did you use hookah at pregnancy? no□ yes□

If yes, average at day: ………. week: ………. month: ……….

1. What was your desire for type of birth: vaginal□ cesarean section□ no matter□
2. What was your husband desire for your type of birth?

vaginal□ cesarean section□ no matter □

1. Have your husband ever done any physical violence against you during this pregnancy?

Often □ sometimes□ never□

1. Have your husband ever done any emotional violence against you during this pregnancy?

often□ sometimes□ never□

1. Have your husband ever done any sexual violence against you during this pregnancy?

often□ sometimes□ never□

1. Did you have feeling of dryness in mouth or was thirsty during active uterine contractions (since having 3 contraction per minute): no□ yes□ (how much time: ………. )
2. Did you eat or drink? no□ yes□ (liquid□ solid□ )
3. Height (CM): ……….
4. Weight at the beginning of pregnancy: ……….
5. Weight in the labour room: ……….
