# Supplementary material for: Pre- and during- labour predictors of dystocia in active phase of labour: a case-control study
Source: BMC Pregnancy Childbirth. 2020 Jul 28;20:425. doi: 10.1186/s12884-020-03113-5 (PMC7388514; doi:10.1186/s12884-020-03113-5)
Supplement: Supplementary file 2 — Additional file 2. Labour progress sheet. [file 12884_2020_3113_MOESM2_ESM.docx]

Forme no 2: Labour progress sheet

Code:

| Exam Time |  |  |  |  |  |  |  |  |  |
| --- | --- | --- | --- | --- | --- | --- | --- | --- | --- |
| Dilatation |  |  |  |  |  |  |  |  |  |
| Effacement |  |  |  |  |  |  |  |  |  |
| Presentation |  |  |  |  |  |  |  |  |  |
| Position |  |  |  |  |  |  |  |  |  |
| Decent |  |  |  |  |  |  |  |  |  |
| Amnion sac(tact/intact) |  |  |  |  |  |  |  |  |  |
| Count of uterus contraction per 10 m |  |  |  |  |  |  |  |  |  |
| Fetal heart rate |  |  |  |  |  |  |  |  |  |

**Pelvic exam Results**

Schial spine: ……….

Pubic arch: ……….

Promontoar situation: ……….

Concavity of sacrum: ……….

Amnion sac status: normal□ oligo-hydramnious□ poly-hydramnious□

**Vital sign**s

Blood pressure: ……….

Pulse: ……….

Respiratory rate: ……….

Induction: no□ yes□ (if yes, reason of induction: ………. )

Induction with: oxytocin□ (duration of infusion: ………. ) folly catteter□ misoprostol□

Augmentation: no□ yes□

Hyper stimulation of uterus: no□ yes□

Analgesics drug: no□ yes□ (hyocin□ promethazine□ petidin□ )

Remifentanil administration: no□ yes□

Delivery type: vaginal yes: no: ( episiotomy: yes□ no□ )

C-section□ Reason of C-section:

Delivery staff: gynecology resident□ midwifery student□ midwife□ intern□

Breastfeeding: ……………. minutes after birth
