# Supplementary material for: Pre- and during- labour predictors of dystocia in active phase of labour: a case-control study
Source: BMC Pregnancy Childbirth. 2020 Jul 28;20:425. doi: 10.1186/s12884-020-03113-5 (PMC7388514; doi:10.1186/s12884-020-03113-5)
Supplement: Supplementary file 3 — Additional file 3. Neonate form. [file 12884_2020_3113_MOESM3_ESM.docx]

Neonate form

1. Cardiogram (admission)

Normal ⭘ Late deceleration ⭘ Variable deceleration ⭘

1. Cardiogram during labour:

Normal ⭘ Late deceleration ⭘ Variable deceleration ⭘

1. Amnion fluid color: Clear ⭘ 2. Meconial ⭘
2. APGAR score at minute 1: …………..
3. APGAR score at minute 5: …………….
4. Need to resuscitation: No ⭘ Yes ⭘

If yes, PPV ⭘ cardiac massage ⭘ drug ⭘

1. Admission at NICU: No ⭘ Yes ⭘

If yes; why:…… Duration: ……..

1. Weight of neonate:……….
2. Height of neonate:……….
3. Head circumcision of neonate:………
